# An Essay on Clinical Instruction

**Published:** 1836-04

**Authors:** 


### Art. III.

1. *An Essay on Clinical Instruction, by* P. Ch. A. Louis, m.d. [*Translated by* Peter Martin, Member of the Royal College of Surgeons, London.—1835. Pp. 33.

2. *Recherches sur les Effets de la Saignée dans quelques Maladies Inflammatoires, et sur l'Action de l'Emétique et des Vésicatoires dans la Pneumonie.* Par P. Ch. A. Louis, Médecin de l'Hôpital de la Pitié, &c.—Paris, 1835. Pp. 120.

*Inquiry concerning the Effects of Bleeding in certain Inflammatory Diseases, and of the Action of Emetic Tartar and of Blisters in Pneumonia.* By P. Ch. A. Louis, Physician to the Hospital of La Pitié, &c.

3. *De l'Emploi du Tartre Stibié à haute Dose dans le Traitement des Maladies en général, et dans celui de la Pneumonie et du Rheumatisme en particulier.* Par Alm. Lepelletier de la Sarthe, Professeur de Pathologie et de Physiologie, &c.—Paris, 1835. Pp. 224.

*On the Employment of Emetic Tartar in large Doses in the Treatment of Diseases in general, and in that of Pneumonia and Rheumatism in particular.* By Alm. Lepelletier de la Sarthe, Professor of Pathology and Physiology, &c.

It gives us great pleasure to have an opportunity of noticing, from the French school, attempts to improve directly the therapeutic branch of our art,—that department which the public will ever regard, and medical men ought to regard, as the most important. We are aware of the great improvement which the treatment of diseases necessarily derives from precise ideas regarding its nature, and how much this precision has been augmented by the pathological labours of our ingenious and indefatigable colleagues on the other side of the Channel. It should however be remarked, that the advantage in a curative point of view hence resulting, has been almost entirely limited to the accurate distinction between inflammation and changes in organs not originating in this morbid condition; and, consequently, to the employment of the remedies of inflammation in certain cases, with that degree of vigour which a perfect knowledge of its existence imparts, and in the abstinence from them in other instances, in which the same accurate knowledge teaches us they would be superfluous and injurious; but, we regret to add, with the suggestion of no means of controlling these non-inflammatory conditions, or, at least, of none possessing a probability of beneficial results calculated to win for them the confidence of the medical community.

This we believe to be briefly our present position in all cases of danger; all which threaten immediately the duration of human existence: we think only of inflammation, seek only for inflammation, and treat only inflammation: at least, we treat this alone with decision and vigour; out of this line, our movements are groping and hesitating.

At a superficial glance, this might appear a humiliating view of medical science and practice; but when we reflect how much of disease is in reality inflammation, the accurate culture of this limited but very extensive domain will be justly regarded as no inconsiderable achievement of art: "est quiddam prodire tenus." We would remark, moreover, that there is a growing feeling among medical men of the propriety of proceeding, if possible, beyond these limits. This disposition is discernible rather in the measures of practical men at the bedside, than in the productions of the press; though, in the writings of the late Dr. Gooch, Dr. Marshall Hall, and others, indications of its growth may be discovered; for in medicine, as in other human pursuits, the press is often but the echo of public feeling. We do not know, however, that these attempts to add, in a remedial sense, to our knowledge of acute disease have proceeded beyond the accurate discrimination of neuralgias from other maladies of a more dangerous nature, which they occasionally simulate, and the recognition of asthenia and anæmia as actual *existences*, and sources of danger. The latter congenerous affections, the dread of which constituted, little more than a quarter of a century ago, the overwhelming feeling of the profession, "like Aaron's serpent, swallowing all the rest," and which subsequently fell into a neglect as unreasonable as their former superstitious worship, may be regarded, however, in their resuscitation, rather as the instruments of improving our treatment of inflammatory disease, than as opening out perfectly new and distinct views of practice.

Many of us may recollect the day when the treatment of inflammation was absolutely bad on both sides of the Channel; our Gallic neighbours erring from the excessive dread of asthenia to which we have just adverted; ourselves from regarding the lancet as the sole remedy, and employing it with a degree of rashness that seemed occasionally irrespective of the present safety of the patient, and always of his future health and comfort. A grievous error, if we mistake not, lay at the root of this Sangrado-like practice. It was conceived possible instantly to extinguish inflammations, and general bloodletting was considered the specific by which this extinction was to be accomplished. Consequently, if the first operation, however abundant, did not attain this object, enough of blood had not been drawn, and a second, a third, a fourth, &c. were resorted to, however slight might be the indications of local affection yet remaining, and how much soever of the apparent constitutional excitement might be, in truth, but a degree of irritable, feeble reaction, which most practised eyes have remarked from excessive depletion. The fact we believe to be, that no amount of depletion,—indeed, no remedial measures, however violent,—will instantly cure inflammation. Our remedies abate the constitutional excitement, and, if they do not diminish the local affection, are a check on its increase. By thus keeping the disease within the sanative powers of the constitution, they diminish its duration, and conduce to the safety of the patient; but do not, in any case, instantly extinguish the affection. This is the information we have often received from the stethoscope in pneumonia, and we have witnessed effects from bleeding in visible inflammation which tend strongly to confirm it. When, some years ago, ophthalmia prevailed in the army, patients were often bled to syncope, losing sixty or seventy ounces of blood. The conjunctiva partook of the general paleness, being pearly white during the fainting fit; but, on the circulation being restored, the vessels were again gorged with blood, and the disease was still to be treated.

The first step towards a rational practice in inflammation,—towards that middle path, equally remote from continental timidity, which allowed the patient to die, and that insular rashness which killed him,—was the employment of mercury as a substitute for further depletion in the advanced stage of the affection. The next we owe to the French pathologists, and more particularly to Laennec, who, by endowing us with the means of precisely ascertaining the site of certain inflammations, induced us to substitute leeches and other local measures in the vicinity of the affected part for a proportion of our general bleedings, and thus to do something towards obviating that anæmious condition, of which the older members of the profession have all of them witnessed such melancholy examples.

How far the remedy, *Emetic Tartar* in large doses, which forms the sole subject of one of the important works now under review, and is ably discussed in another, may conduce to the same desirable result, is a question well worthy of consideration; and certainly the able authors, in a different mode, but each with much talent, have furnished copious materials for attaining a correct conclusion. Indeed, we have much pleasure in observing that our neighbours are displaying in therapeutics the same minute and accurate investigation of facts, and the same patience in recording them, which have shone so conspicuously in their pathological pursuits.

A very imperfect idea would be given of the work of that truly philosophic physician, M. Louis, did we consider it without reference to the method of close analysis and cautious induction which it is his object to introduce into medicine, and of which the work under consideration may be considered as the first therapeutic fruits presented to the public. This method has been embodied in a pamphlet, not originally intended for publication, but of which a translation has been published (with the permission of the author,) by Mr. P. Martin, surgeon, of Reigate; and from this small work we shall extract a succinct account of the system, both for its value, and in order that the reader may have it in his power to judge how far M. Louis has, in his therapeutic work, made a successful application of his own "Organum." We must observe, that Mr. Martin's translation of this little work of M. Louis presents a pleasing proof of the impression produced by M. Louis on his pupils, including those of our own country. Not a few of them, probably, like Mr. Martin, will ever acknowledge, and gladly acknowledge, that the strongest impulse given to their industry, and the most enlightened direction given to their enquiry, originated with a teacher to whom they were only recommended as young foreigners desirous of information. The example of M. Louis can never be lost upon them; and Mr. Martin has given an earnest of this, whilst he has paid a tribute of gratitude to M. Louis, by taking pains to lay this little work before the English reader.

The Essay on Clinical Instruction consists of two divisions; the first treating of the study of particular facts, or of diseases considered in an isolated manner; the second being a summary (*resumé*) of a clinical course, or an enquiry into general facts. In the first division, the clinical professor is advised, previously to studying the symptoms of a disease, to inform himself "of the age and profession of the subject; of his habitual state of embonpoint or of emaciation, of strength or of weakness, of health or of disease; of the affections under which he has laboured previously to the present; and of his good or bad conformation." Valid reasons are assigned by the author for accuracy on all these points, not merely in reference to the individual case, but likewise with the more important view of obtaining statistical data for the advancement of science. These preliminary questions settled, the physician is to endeavour to learn from the patient the precise period at which the disease commenced; and judicious suggestions are given for surmounting the difficulty which we have all experienced of obtaining correct information on this head. He should then proceed to the investigation of symptoms; and M. Louis advises that

"These should be studied successively, one by one, in the order of their development, from their appearance to the moment at which the patient is submitted to our observation, and from this moment to the determination of the malady: the precise period of the appearance of each must be enquired into; without which we might, following the example of more than one author, be induced to consider as primitive, symptoms which are only secondary, and consequently to deceive ourselves with regard to the true character of the affection."

With respect to this part of the subject, symptoms, comprising the results of auscultation, and percussion, and necroscopic observation, he insists on the utmost minuteness.

"Pathological anatomy," he says, "declares the value of the other methods of exploration which it interprets. Without it, for instance, how should we know that crepitous *râle* indicates the first degree of pneumonia; that *gargouillement* and pectoriloquy demonstrate the existence of tuberculous cavities? that ægophony corresponds to an effusion of fluid into the cavity of the pleura? the symptoms of softening of the brain to that affection? those of adynamic fevers to a profound alteration of Peyer's glands? Evidently these facts would be unknown to us.

"We might certainly, without the assistance of this science, and by the aid of symptoms alone, determine the seat of a great number of maladies, but of their nature we should remain ignorant. The symptoms of apoplexy indicate sufficiently that the brain is the seat of this affection, it is the same with the symptoms of softening of this organ; but how, without the aid of pathological anatomy could we know that the former are caused by a hemorrhage, the latter by a softening or inflammation. But as the history of symptoms becomes valueless unless they have been noted with great care, from their commencement to their termination; unless all the functions have been interrogated in the same manner; so pathological anatomy can only render to science all the service which might be expected from it, when we proceed with extreme care to an examination of *all* the organs in those cases which have proved fatal."

M. Louis subsequently informs us, that "its object is not merely to point out the seat of diseases, to shew their nature, to verify or unravel their complications: it is the only means of arriving at the knowledge of a great number of the laws of the economy of disease which are the most important; and this knowledge can only be the result of an equally attentive examination of *all* the organs." Certain of these laws, brought to light by the philosophic industry of the author, are explained and exemplified in the essay before us, but much more fully in his Treatise on Phthisis; an account of which, translated by Dr. Cowan, is given in our first Number.

Causes, including hereditary predisposition, are to be investigated with the greatest care; and suggestions are given for conducting the investigation. The author would reject all statements unaccompanied with that full detail of circumstances fitted to substantiate the facts. To be told that a man's father has died of phthisis is nothing, unless the individual making the declaration can describe symptoms which prove this to have been the fact. The evidence, however, in cases of this sort being generally derived from non-professional witnesses, and treating of the circumstances and feelings, not of the witnesses, but of others, we think that the instances will be rare in which it can be obtained of the degree of minuteness required by M. Louis. In vindication of the preciseness demanded both on this and other points, he makes the following sound remark:

"These details, I am convinced, will not appear too much extended to those who remember that medicine as a science rests entirely on observation, and that particular observations are to the physician what experiments are to the natural philosopher: that if the latter cannot be too careful and exact in his experiments, it is the same with the former, in reference to his particular observations, to the manner of interrogating his patients, of investigating their state, and the causes which may have contributed to the development of their diseases."

The task of examination completed, the reasoning follows. We must convert our symptoms into signs, as through them we are to arrive at a knowledge of the organ affected, or of the pathological state of the subject; a task in ordinary cases of no great difficulty, but in others presenting real difficulties, to be subdued either by considering the series and connexion of the symptoms, or by way of exclusion; that is, by observing that one organ only being affected, the disease must be *there,* however slight the affection may apparently be; or by recurrence to a well-established law in the economy of disease, to which we have already alluded, and which M. Louis himself was the first to develope,—that a certain alteration, or a certain evident disease, supposes the existence of another still latent.

"But, having discovered," he adds, "the organ affected, itis necessary also to determine the disease of which it is the seat: a new, and in the present state of the science, sometimes an insurmountable difficulty; not so much from the divergence of the opinions of authors on this point, as from the want of exact and numerous observations in a great number of diseases, from which information might have been derived; especially from the want of anatomical inspections carefully conducted at different periods of the same disease."

If all the preceding information has been carefully collected, the prognosis flows from it almost as a necessary corollary. The author's opinion as to the principle which should guide us in the treatment admits of an equally brief description. He advocates, as might be expected, the empirical method. "Rational medicine," he observes, "is the medicine of experiment: to observation and experiment alone does it belong to pronounce upon all that regards pathology and therapeutics, especially therapeutics, which we have been accustomed to consider, we know not why, as a simple corollary of pathology."

In the second division, we are advised to consider as exact only such facts as have been collected according to the principles, with all the caution and in all the details expressed in the first division; and our analysis should comprise only those facts which have been carefully collected, and as to the accuracy of which no doubt can be raised.

"These facts should be formed into groups: those should be united which by their similarity indicate the same affection; those should be separated which offer opposite characters: and for this purpose, we should not only consider the symptoms in themselves, but also their progress, duration, order of succession, and the different circumstances under which they are developed." * * * "We may be more confident in the classification of facts, when, among those of the same species, there are some relative to cases which have proved fatal. Then there can be no doubt of the similarity or identity, if the same lesion is constantly observed in all, which by its nature and its seat, will explain in a satisfactory manner the first symptom observed.

" This lesion, then, ought to be considered as the anatomical character of the disease; and as it accounts for the first symptoms, it appears they were connected from their commencement with an appreciable alteration of texture, which as I have observed above, is not the case in all diseases.

" The facts being thus classed, it is necessary to study them, and as in each case, or in each disease, the state of all the functions and organs has been ascertained, we should now follow the same function, the same symptom, the same organ, through each particular fact. For in the same disease we observe not merely symptoms or lesions peculiar to the organ affected; but also many others, which are met with more or less frequently, in the most different diseases, and without which we should evidently have but a very imperfect idea of the disease.

" But to have exact data on these points, to know with precision the value of each symptom in a disease, we ought in the first place to seek for the proportion of cases in which it is observed, and this is to be done by *counting.*

" For the words" *more or less,* " consecrated by custom, signify, it will be agreed, either nothing or very little. When it is said, for instance, that a symptom is frequently observed in a disease, does it mean that it is observed, twenty, thirty, forty, sixty, or eighty times in a hundred! Evidently it is uncertain, an expression is used the meaning of which is not known, and which it is not possible to replace by one more exact, except by *the method of counting.* Thus, it was known that diarrhœa was common during the course or at the commencement of typhoid fevers; but to ascertain the real meaning of this word *common,* it was necessary to count; and it is only after having done so, after having ascertained that diarrhœa occurs in two-thirds of the cases, at their commencement, that this symptom has become of great importance in the history, and especially in the diagnosis, of fevers. The same may be said of the rose-coloured lenticular spots observed in the same disease: little notice was taken of them in its history, until it was ascertained that they appeared almost constantly, so as to be wanting scarcely twice in a hundred cases. So that, if one of these two symptoms, the diarrhœa or the typhoid maculæ, were wanting at the commencement, or in the course of an affection, which should otherwise in some points resemble typhous fever, without having the characteristic symptoms of any other disease, we should almost entirely lay aside the idea of typhus." (P. 21.)

This numerical proceeding, which is the distinguishing feature of the author's method, and which, in the passage just quoted, he has applied to symptoms, he extends to all the other circumstances connected with diseases. Their duration; the influence of age, sex, temperament, and treatment, on this duration; their comparative frequency and mortality; the variations of the mortality according to age, sex, season of the year, various localities and climates, and in the same locality at different periods; the proportionate success of different modes of treatment:—all this cannot be ascertained, he conceives, (and we think him correct in the opinion,) otherwise than by counting.

The numerical method admits an important application to the illustration of the causes of disease, which the author exemplifies in the following important remarks:

"It is an opinion still very generally prevailing, that tubercles of the lungs are the result of inflammation of the bronchi, or of the parenchyma of the organ in which they are disseminated.

"But ought not that physician to feel himself much shaken in his opinion, who learns that bronchitis, at least in the severe form, is more frequent in the male than in the female, in the proportion of three to one; that it is the same with peripneumony; whilst phthisis, on the contrary, is less frequent in the male than in the female?

"Similar remarks may be naturally applied to cancer. This disease can scarcely be considered as a termination or consequence of inflammation, by him who knows, from the inspection of a great number of bodies, that whilst pulmonary and intestinal inflammations are the most frequent, cancer appears most commonly in the uterus and stomach; that the liver is next in frequency, and then, at a considerable distance, the lungs and kidneys; that of eight hundred subjects whose viscera have been examined with care, only two examples of cancer of the rectum have been found, and not a single case of that affection in the small intestine." (P. 27-28.)

The reader is now in possession of a faithful outline of M. Louis' method of clinical instruction. We anticipate the remark it will call forth: this is the "Novum Organum" applied to medicine, with such modifications and peculiarities as the nature of our pursuits demands. The remark is just, but at the same time the best eulogium of the system. Why is medicine,—why especially is therapeutics, (its most important yet weakest point,)—in so inefficient a state, but because, with the worship of inductive philosophy on our lips, our hearts have been far from it. Observation in medicine, to furnish us with the general facts we require, must not be limited to a few objects. We cannot reason safely from individuals to individuals, but only from groups or masses of facts to other groups or masses. This arises from the variable character of the phænomena with which we are conversant, and which we cannot modify, as a chemist does his experiments, to our will, but must accept as nature presents them to us; and hence comparisons between individuals are fallacious: but, when instituted between numerous groups, the varieties of which mutually compensate each other, general principles, deserving of confidence and adapted to future purposes, may be deduced. We admit that a professional man, hurrying from street to street of our vast metropolis, or tiring himself and his horses through an extensive rural district, is ill-situated for observing, grouping, and analysing facts in the way required for the "augmenta scientiæ." A certain mental process of the inductive kind he is probably daily carrying on, by which his own knowledge is increased; but he has not leisure, and probably he does not possess the habits of mind requisite, for presenting it to the public in that statistical form which alone can carry conviction to the mind of others. When a new remedy is proposed, a *trial* is given to it with greater or less confidence, according to the authority of the individual proposing it: it is never employed at once, with a certainty of its beneficial effect, from the amount of well-sifted, positive, and comparative evidence, by which it is supported. We are all insulated, each man gathering for himself, or, if he sometimes listens to the suggestions of others, it is with suspicion and hesitation. Some become wise and skilful; but, when they die, their wisdom and skill perish with them. What we lack are principles, or general facts, reposing on sufficient evidence to convince us of their validity, communicable to all, and available for the purposes of all. Such principles, if we are to obtain them, must emanate from great public institutions: and for forming them we can recommend no surer guide than M. Louis.

We now proceed to present the reader with a view of M. Louis' therapeutic work on the effects of certain remedies in inflammatory diseases.

The first article treats of bloodletting in pneumonia, and its purport is thus stated by the author:

"The subjects of which I am about to study the history are seventy-eight in number. Of these twenty-eight died, and all were in a state of perfect health at the period when the symptoms of the disease displayed themselves.

"Of the fifty individuals who recovered, three were bled on the first day of the affection, as many on the second, six on the third, eleven on the fourth, six on the fifth, five on the sixth, six on the seventh, as many on the eighth, and four on the ninth; and the mean duration of the affection was, in the order indicated, of 12, 10, 18, 19, 22, 20, 12, 17, and 23 days. But the following table will make more clear the relation between the duration of the affection and the period at which the first bleeding took place."

Of this table we copy only the final column, or that comprising the sum of the details; premising that the upper figures indicate the day when the first bleeding took place; those on the left, in the final column, shew the duration of the disease; those on the right, the mean number of bleedings employed.

| 1 | 2 | 3 | 4 | 5 | 6 | 7 | 8 | 9 |
|---|---|---|---|---|---|---|---|---|
| 12 $2\frac{1}{3}$ | 10 $2\frac{2}{3}$ | 18 3 | 19 3 | 22 3 | 20 $2\frac{3}{5}$ | 19 $2\frac{1}{3}$ | 17 2 | 23 2 |

"That is to say," continues the author, "if we could establish a general proposition by the aid of this small number of facts, we should inevitably conclude that the antiphlogistic treatment, undertaken during the first two days of an attack of pneumonia, has the power to abridge its duration very much; but, these two days having elapsed, that it is of slight import, whether he commence it a little sooner or a little later. But the sort of contrast which exists between these two propositions ought to make us suspect their correctness, and a thorough examination of the facts shows, in effect, that the influence of bloodletting, performed during the first two days of the disease, is less than it seems to be at first glance, and that in general its power is very limited.

"In cases recorded in the same column, or in which the antiphlogistic treatment began on the same day (independently of those in the first and second columns), the duration of the malady presented the greatest variations, so that in those of the fourth column, some were convalescent on the twelfth day, others (not to take extreme terms) on the twenty-fifth or twenty-eighth," [his table would have justified his saying the fortieth,] "which cannot be attributed to the degree of the disease, which was the same, or to the difference of treatment, which was equally energetic, and directed by the same physician. Whence it seems to result that, in these cases the utility of bleeding had very narrow limits.

"Differences not less considerable in the duration of the affection would without doubt have taken place in the individuals bled during the first twenty-four or forty-eight hours, had their number been greater. And, on the same supposition, the difference of the mean duration of pneumonia, in the subjects bled the two first days and in those who were not bled till a period more remote from the commencement, would have been on the contrary less considerable. So that we should approach nearer the truth, we should discover better the real difference effected in the course of the disease by the greater or less promptitude with which the bleedings were performed, by taking the mean duration of the disease, on the one hand in the cases bled the first four days; and, on the other, in those who were not bled till the period from the fifth to the ninth inclusive. And then the mean duration of the pneumonia would be *seventeen days* in the first, and *twenty* in the second class." (P. 8-12.)

After shewing that faults in diet were committed by those who were bled late in the disease, which render his estimate too favorable to those in whom the operation was performed early, M. Louis proceeds to disprove the existence of certain supposable sources of fallacy in his data, which might have led him into an opposite error. He shows, in fact, that a difference in the ages of the two classes of patients,—a variety in the intensity of the disease,—an error in calculating its actual commencement and termination; or the operation of other remedies, such as blisters, did not in the least tend to vitiate his conclusion. "It remains, then," he says, "demonstrated that bleedings have had but a very limited effect on the course of pneumonia, in the cases which occupy us." (P. 16.)

"Facts relating to the fatal cases confirm these conclusions, and appear to contract still further the limits of the utility of bloodletting. In effect, of the twenty-eight individuals in question (the fatal cases) eighteen were bled during the first four days of the disease, nine from the fifth to the ninth; and if we unite, on the one hand, all those who were bled for the first time during the early period of the pneumonia, whatsoever may have been its termination; on the other, those who were bled later: we have, on the one part, in the order indicated, forty-one cases, of which were fatal eighteen, or about three-sevenths; on the other, thirty-six, among whom nine, or the fourth part only, underwent the same fate—a result frightful, and in appearance absurd." (P. 16.)

In explanation of so astounding a result, which seems to shew that bleeding is a noxious, not a beneficial agent in the disease, he presents us with a table. This, however, we omit, finding his verbal clearer than his tabular illustration. "We see, in fact," he adds, "that the patients bled the first four days of the disease were older than those on whom the antiphlogistic treatment was not employed till after that period, in the proportion of fifty-one to forty-three years; a difference which, without being very considerable relatively to its presumed effect, might have a great influence on the issue of the disease." He says, moreover, that, in summing together all the patients treated, whether the result was fatal or otherwise, the difference was still less, being as forty-one years to thirty-eight; but we learn that the number of those in the first class whose ages exceeded fifty, was nearly double that of individuals of the same age who were bled later, which might have a great influence on the mortality. In this view we concur, having ever found pneumonia a very fatal disease in aged subjects, but comparatively tractable in youth.

Passing over two articles, one comprising cases illustrative of the effect of bloodletting on erysipelas of the face, the other on cynanche (angine gutturale), with the remark that this effect, if any existed, was extremely small, we follow our author to another group of cases of pneumonia; hoping, by concentrating the reader's attention on one disease, and that the one, of all others, in which antiphlogistic measures are considered as the most necessary and influential, to enable him to form a clearer idea of the method of investigation, and of the results to which it may ultimately lead.

These cases were twenty-nine in number: four were fatal, whilst twenty-five recovered, and quitted the hospital in perfectly good health. The patients were healthy immediately prior to the occurrence of the symptoms, and the symptoms were of a nature to place the existence of the disease beyond all question.

"Of the twenty-five who recovered," says M. Louis, "none was bled on the first day of the disease. The first bleedings were performed on the second, third, fourth, fifth, sixth, and seventh days of the affection, with one exception only, that of an individual whose convalescence took place on the twenty-second day, and who was not bled before the fourteenth. The disease lasted, on the average, observing the order indicated, fourteen, eighteen, fourteen, fourteen, fifteen, nineteen, eighteen, and twenty-two days. That is to say, that at the first glance it would appear quite indifferent, whether patients seized with pneumonia were bled for the first time, or the second, fourth, or fifth day of the disease, since its mean duration was very nearly the same for the three groups of cases bled at these different periods. Nevertheless, on summing together, on the one hand, those who were bled for the first time, from the second to the fourth day inclusive; on the other, those who were so afterwards, we find that the mean duration of the disease was fifteen days and a half for the first, and eighteen and a quarter for the second group. Whence it would appear a natural conclusion, that the influence of bleeding performed at a period more or less near to the commencement of the disease was a little more marked in the cases in question than in those of which the history forms the subject of the first chapter, and in which the mean duration of the disease was seventeen days and a half, and twenty days." (P. 35.)

It is to be observed, that there is a discrepancy here between the estimate of the mean duration of the disease in the early-bled cases of the first group, and that which we translated literally from page 12. The author's statement *then* was seventeen days; he now rates it at seventeen and a half; and, on summing up and dividing the figures of the first table, we find the latter statement the correct one. The reader will not think this remark superfluous, when he reflects that, had the first calculation been correct, the whole amount of difference being but a quarter of a day, the advantage of early bloodletting would have been a little more manifest in the *first* than in the *present* group, the conclusion of M. Louis being thus reversed. Since observing this discrepancy, we have re-examined all the calculations of which the results are presented to the reader, and find them accurate (with the exception mentioned,) saving some fractions, represented with sufficient correctness by the approximating terms, one half and one quarter.

The question may be raised, and with justice, to what extent is the duration of a disease the measure of its intensity and danger? The author has thus far shewn that the influence, over this duration, of bloodletting performed early, is not much more considerable than that of a later bleeding; but he has shewn no more. A disease may be slow and lingering, and yet not productive of more suffering, or attended with greater danger, than a malady more acute and rapid. We constantly observe, for instance, that in any given epidemic of fever, of which the general course is long and tedious, the individual cases are milder, and the mortality is less, than in a disease which reaches its goal more quickly. The author himself admits, with regard to pneumonia, that the duration of the disease is not a measure of its severity, when he says (p. 11,) that a difference of as many as thirteen or sixteen days in the period at which certain patients became convalescent did not arise from a variety in the degree of the affection, "which was the same in all." What we all wish to acquire are precise ideas regarding the influence of remedies on suffering and mortality; and we should consider the extent to which the duration of a disease is affected by them to be an imperfect means of calculating this influence. A much more correct estimate of the effect of bloodletting may be formed, we conceive, from M. Louis' account of its operation on the general state and particular symptoms of patients in pneumonia, which we regard as one of the most interesting parts of this valuable little volume. He has minutely detailed the modifications which the symptoms underwent under the influence of bloodletting in both groups of cases,—the seventy-eight which form the subjects of the first chapter, and the twenty-nine which are discussed in the present one. For the sake of brevity and clearness, we shall confine ourselves to the second collection of cases, indicating, as we go on, any discrepancies in the effects of the two groups. We are induced to select the second group, the rather that the practice adopted regarding them bore a greater resemblance to that habitually employed in similar cases in this country, than was observed in the treatment of the subjects of the first chapter; the bleedings, in the former case, having ranged in quantity from twenty to twenty-five ounces each, or even to syncope, and in number from one to three: those in the latter having been, in quantity from ten to fifteen ounces, and in number from a single bleeding even to seven.

After pointing out the impossibility of effecting an instant cure of pneumonia, M. Louis remarks:

"The circumstance which has imposed on practitioners, and induced them to believe that it was easy to extinguish pulmonary inflammation at its commencement by means of large bleedings, is the fact, that in some cases, in truth not very common, bleeding performed at this period, is followed by a considerable amelioration of the general symptoms, and of some local symptoms, namely the pain and the dyspnœa. But the other phenomena remain, and even increase in intensity and extent after the first bleeding, if it has taken place *near the beginning of the disease;* and if we do not then examine the patient with care, we believe that we have extinguished a disease of which we have only considerably diminished the febrile movement and some other symptoms. I observed last year a remarkable example of this fact, in the case of a young man, admitted into la Pitié within twenty-four hours after the first invasion of illness, and having then all the symptoms of pneumonia; extreme dyspnœa, much pain in the left side, hurried respiration, rapid pulse, and great heat. He had orthopnœa, the sputa rust-coloured, viscid, and semi-transparent, the sound of his thorax a little obscure behind and inferiorly, where were heard, at the same time, a crepitating râle, a respiration confused, or, as it were, bronchial in some points, and broncophony without egophony. He was bled from the arm to syncope soon after his arrival, and lost twenty-five ounces of blood. Soon after, he experienced great relief; and on the following day many persons, who were present at my visit, thought they had before their eyes a pneumonia extinguished. The pain was less than in the evening; the pulse was but a hundred; the anxiety had disappeared; and the expression of the physiognomy was natural. Nevertheless, the sputa preserved their characteristic appearance, and the obscurity of sound, and the broncophony existed over a more considerable space than in the evening. So that the pneumonia, far from having been instantly cured by a first and copious bleeding, had acquired after it a greater development and extent: a development which did not stop till the fifth day of the disease, of which the convalescence did not commence till from the ninth to the tenth." (P. 38.)

The great relief afforded in this case, (and we could furnish from our own records many parallel instances,) thougn the local affection not only remained, but even extended, we believe to be explicable mainly on a general principle not adverted to by our author, but of which innumerable examples have proved to us the existence,—namely, that dyspnœa, when not depending principally or solely on spasm of the bronchi, is materially relieved by the prompt diminution of the circulating fluid. Not only in inflammation have we constantly witnessed this, but in affections of a different character, particularly in valvular disease of the heart; in which we have seen the patient in a state of orthopnœa, with a swollen and suffused countenance, injected eyes, and expanded nostrils; and then, shortly after a full bleeding from the arm, in a state of comparative ease and comfort, extended composedly on his bed. We are not disposed to detain the reader with reasonings on the expediency of adapting the amount of the circulating fluid to the extent of lung left free to render it arterial; or by running the parallel between the condition of this organ in pneumonia and when the pulmonary vessels are gorged with blood, in consequence of the difficulty of its transmission through the heart; but we advance the general fact as one of practical value. Not only does it furnish an explanation of the relief afforded in the case quoted by M. Louis, and in similar instances, but it appears to explain why, in all ages and countries, venesection has been so generally adopted in the treatment of inflammation of the respiratory organs. In pleurisy, says Celsus, "remedium est magni et recentis doloris sanguis missus;" in peripneumony, he remarks, "Oportet, si satis validæ vires sunt, sanguinem mittere;" and the tone of all the ancient writers is the same. The effect on one of the most urgent symptoms being immediate and manifest, it is natural that it should be a remedy of general adoption in the infancy of the art, and should retain its reputation through a succession of ages; but the same circumstance is a reason for our studying minutely the nature and extent of its action, that we may acquire the greatest possible skill in its application. We have, therefore, great satisfaction in laying before the reader so full an account of its effect on the individual symptoms of pneumonia as is contained in the following extract:

"The pain was not removed in any case by the bleedings; it was even but very little modified by general bleeding, twenty-four hours after which it was found only a very little less severe than on the preceding day, in the majority of cases. It still existed on the sixth day of the disease, in a subject on whom the operation was first performed on the second; who lost in forty-eight hours fifty ounces of blood by the lancet, and from five to six ounces on the fourth day by the application of twenty leeches to the painful points. Its mean duration was seven days and a half, both in cases of inferior pneumonia and in those in which the disease affected the superior lobe;* and in one of these last cases, in which

* In a long note here, the author rather explains a fact stated by M. Andral, than differs from that accomplished pathologist. M. A. had remarked that pneumonia of the the patient was bled even to syncope and lost thirty ounces of blood on the third day of the affection, the pain was only a little diminished on the following day.

"Respecting the first group of cases, it is mentioned that the pain generally increased during the twelve or twenty-four hours immediately following the bleeding: it is stated, likewise, that it yielded more quickly to local than general bleeding. In other respects, the statements regarding the pain in the two groups correspond.

"The sputa did not lose their pathognomonic character in any case on the day following the first bleeding; not even in those cases which have just been quoted, and in which the sanguineous evacuation was very copious. The duration of this character was proportional to that of the disease, so that the *sputa* did not cease entirely to be characteristic, on the average, till the seventh day of the pneumonia, in those who were bled before the fifth: and on the ninth in those who were bled later; and, as was remarked in the cases of the first chapter, the influence of losses of blood on the sputa appeared the more marked in proportion as they were performed for the first time at a period more remote from the commencement, so that in the individuals bled late, or after the fourth day, the sputa showed nothing remarkable twenty-four, forty-eight, or seventy-two hours after the first bleeding; whilst, in those who were bled early, their pathognomonic character never completely disappeared before three days, counting from the first bleeding. A difference which can only be explained, as I have said, by the circumstance of the disease approaching its natural term in the former cases, and being more or less remote from it in the latter.

"Besides, if the duration of the affection was very variable in individuals bled the same day, this was equally the case with the characteristic appearance of the sputa, which continued, in different degrees, during a space of time varying from four to eleven days, or from seven to fourteen, in cases bled for the first time, before or after the fifth day."

It is remarked concerning the influence of bleeding on the *sputa*, in the first group of cases, that "their pathognomonic character became more apparent (*saillante*) if the loss of blood took place at a period near the commencement of the malady. It became much less evident, on the contrary, the day following the bloodletting, in those for whom it had been prescribed at a remote period:" facts which he explains, as in the instance of the first group, by the disease approaching its natural termination in the latter case, and being remote from it in the former.

"The *crepitation* remained longer than the two symptoms already named; from ten to fourteen days, in the cases bled before the fifth day; upper lobe was more serious than that of the lower. M. L. regards this as a simple coincidence, and pneumonia of the upper lobe as more severe in appearance *only*, because it is the pneumonia of old men. In fact, the third part nearly of M. Louis' cases were affected with *superior* pneumonia, and their average age was fifty-four; whilst the mean age of those in whom the inferior lobe was inflamed was thirty-five; and, moreover, of these cases one only was fatal.

From the same circumstance it arises that superior pneumonia in those who recover exceeds the affection of the inferior lobe in duration by three days; a difference, he adds, which is nearly the same for each particular symptom.

from ten to nineteen in those subsequently bled; mean term, twelve days for the first, and fourteen for the second. It was not at once extinguished in any case.

" *The respiratory murmur* was more or less considerably affected during eighteen days, as a mean term. The modification of this murmur, designated by the phrase bronchial respiration, did not yield in any case to the first bleeding; and it was the more influenced by the employment of this means, as it was more tardily adopted: so that, it was much less perceptible the day following a bloodletting performed on the sixth day; and that an analogous diminution did not take place in individuals bled for the first time on the second or third day of the disease, till three days after the bleeding.

"The *broncophony*, which arises from the same causes as the bronchial respiration, followed the same course, and had the same duration.

"The *obscurity of sound* existed in all the cases, and remained, on an average, till the nineteenth day of the disease; its diminution being gradual: and if we except two cases bled on the fourth day of the disease, in which the obscurity of sound was much less the day following the opening of a vein than the preceding day, this amelioration did not commence till from two to five days after the first venesection, and so much the more tardily in proportion as this operation was performed at a period nearer the commencement.

" In three cases in which the bleeding was performed on the second day of the disease, the *pulse* fell the following day from 120 and 100 to 108, 80, and 96. But the day following a second bleeding, it was 104, 108, and 90: that is to say, it had fallen, after two bleedings, only a few pulsations.

" It was the same in the individuals bled on the fourth day for the first time, for in them the amelioration was null or momentaneous the following day. But in the great majority of the cases in which the first bleeding did not take place till after the fifth day of the affection, the pulse was less accelerated from the following day, and this amelioration went on increasing afterwards.

" In the cases in question, as well as in those which were analysed in the preceding chapter, venesection did not influence in any considerable degree the course of the symptoms of pneumonia, excepting when it was performed at a period sufficiently remote from the origin of the malady; and, without doubt, as I have already remarked, it then produced an effect, because the disease had approached more or less its natural termination; whilst it was more remote from it in the cases wherein the bleedings took place sooner. And these facts, as well as those relative to the duration of the pneumonia, prove how limited is the utility of bloodletting in the treatment of this affection." (P. 41.)

On reading the last-quoted sentence and many passages in this extract, the question almost inevitably occurs, if bleeding produces any apparent effect only because the disease is reaching its natural termination, may not the operation and the improvement be mere coincidences, and all the change observed but the natural progress of the disease to resolution? It is admitted that the malady is near its close; the bleeding has been long delayed, and we can hardly help regretting that there is not sufficient hardihood to withhold it altogether: for, till such a measure be adopted, it is impossible to estimate its real value as a therapeutic agent. We see one obstacle, and we fear it will prove insurmountable, to the perfectly successful pursuit of M. Louis' object of testing the importance of our remedial measures,—our total ignorance of the natural course of acute disease. We have two powers in operation: the tendency within the constitution to self-reparation, (*vis medicatrix naturæ,* if the term be not objected to,) and our therapeutic means: we are ever at a loss to know what share of the fortunate issue to attribute to each; and this obscurity, as it appears to us, must envelope our facts and confound our reasonings, till some one shall be found bold enough to intrust the cure of pneumonia, and other serious internal phlogoses, to repose and water-gruel. The homœopathists might have assisted us; for their doses of medicine, which are so infinitessimally small that we lack fractional numbers of sufficient exiguity to express them, might have been disregarded in the estimate; but they have been, apparently, little occupied in combating acute physical disease, or rather in watching and recording its natural progress.

Practitioners in our sea-ports occasionally observe deplorable examples of the ravages of disease in sailors returned from long voyages in merchant vessels, where they are destitute of medical assistance; but similar ravages are observed on shore, where professional aid is in operation; and, moreover, nothing is known of the numbers who may be attacked with severe maladies at sea, and emerge from them comparatively uninjured. In such cases, besides, not only are what we suppose to be, and let us hope correctly, the *juvantia* wanting, but the *lædentia*—errors in diet and regimen—are in full operation; so that no correct conclusions can, as it now appears to us, be drawn from such premises. We have two unknown quantities, the value of both of which could be calculated, were either excluded; but the one, the sanative power ot the constitution, is beyond our control; the other, the action ot remedies, there are feelings in our nature (and those very creditable ones,) which forbid us to exclude, and the problem will probably remain unsolved, excepting that a degree of light may be thrown on it by approximations to the truth, such as those furnished by our author, and others who may follow in his footsteps.

There are several interesting matters in the volume which our limits compel us to leave untouched, or to advert to slightly. For instance, M. Louis applies to the effects of blisters the same scrutinizing analysis that he has directed to the agency of bloodletting, and comes to the conclusion that in pneumonia they are devoid of utility. He allows that he has been induced to exclude them from the treatment of thoracic inflammations in general by certain analogical reasonings, in which we are somewhat surprised to see so severe a logician indulge. These we shall not detail; neither do we think it necessary to transcribe the more valuable statistical facts, which furnish a much better reason for their exclusion from the treatment of pneumonia in particular; but we fully concur in the justice of the following remark: "It certainly is beyond a doubt, and cannot be too often repeated, that we do not know the therapeutic value of blisters; and that it ought to be studied, with the assistance of numerous and well-observed facts, absolutely as if we knew nothing regarding them."

His opinion of opium, both in pneumonia and other affections, particularly cerebral ones, in which general sentiment or prejudice runs strongly against it, is much more favorable; but we hasten to lay before our readers his observations on a subject which in this country we regard as one of greater importance, the employment of emetic tartar in large doses.

The great disproportion in the mortality which took place in the two groups of cases will have arrested the reader's attention;—for, of the first group, or those treated at la Charité, 28 died out of 78, or more than one-third; in the second group, those at la Pitié, 4 out of 29, or less than one-seventh. M. Louis applies himself to the investigation of this immense disproportion, and, after attributing it in part to the bleedings, which were less numerous in those treated at la Pitié than in the first class of cases, but to a greater amount at each operation; and which circumstances, he thinks, had some influence in producing the more fortunate result in the subjects of the second chapter; he thus proceeds:

"To sixteen of the individuals who recovered, emetic tartar was administered, during a period of from four to seven days, in quantities progressively increasing from six to twelve grains in six ounces of the distilled water of the flowers of the linden-tree (eau distillée de tilleul) sweetened with half an ounce or an ounce of syrup of poppies, and the patients took these quantities in six or eight doses. Their disease lasted, on an average, eighteen days; three days longer than that of the individuals not subjected to this treatment: so that it would appear at the first glance, that the emetic tartar had a pernicious effect on the course of the disease, instead of having accelerated its fortunate termination.

"But this influence was pernicious in appearance only. The emetic tartar was administered, after several bleedings had been performed, on the eighth day of the affection on an average, because the disease continued acquiring greater and greater intensity; and in cases not bled for the first time till the fifth day, as a mean term: whilst it had been performed on the third day in the cases in which this medicine was not employed. That is to say, it was given under the most unfavourable circumstances, and in severe cases, which explains the long duration of the disease in those who took it. Let us add, and it is not necessary to insist on the importance of this fact, that the patients, for whom the emetic tartar was prescribed, were older than those who did not take it, in the proportion, on an average, of forty-five years to thirty-one: an enormous difference, which shows that not only had the medicine no pernicious effect on the duration of the disease; but that in some cases it must have accelerated its course and prevented a fatal termination.

"This last proposition appears, moreover, to be confirmed by the changes which almost immediately followed the exhibition of the emetic tartar. From the day following that of its first employment, fifteen of the seventeen persons who took it found themselves a little better, or much better, having then perceptibly more strength, an improved physiognomy, and the respiration less restrained. Besides, thirteen of them, whose chest emitted a sound more or less completely dull over a certain space, when the emetic tartar was first administered, shewed from the following day a perceptible improvement in this respect; percussion of the thorax being already more sonorous: and these various ameliorations were permanent, and made additional progress daily.

"The increase of strength from the day next ensuing, or that in which the medicine was administered, is the more remarkable, as its action was accompanied with frequent purging and vomiting. In sixteen cases out of seventeen, the alvine evacuations were very numerous, ranging from eight to fifteen on the first day, one half less frequent on the second, and, on the third and fourth, not more so than in the ordinary state. The vomitings were less numerous, and of shorter duration, than the alvine discharges: they did not continue beyond the first day, and were absent altogether in five instances.

"Three of the patients who died took the emetic tartar, and did not experience any improvement on the day following that of its administration. One alone of these had not the evacuations mentioned.

"Thus, of twenty cases in which the emetic tartar was employed under unfavorable circumstances, three only were fatal; which cannot leave a doubt, as it appears to me, of the utility of this medicine, in large doses, in the treatment of pneumonia; and so much the more as these three individuals were all aged, being sixty or seventy years old." (P. 51.)

Such testimony, from so calm and cautious an observer, is well calculated to win the reader's attention to the work of M. LEPELLETIER, which is dedicated exclusively to the investigation of the properties of this medicine. The method and style in which M. Lepelletier manages his subject are not unworthy of its importance; his work being learned, copious, and elaborate. In fact, this author errs by excess rather than deficiency. He tells us every thing; a habit in which French systematic writers frequently too much indulge. We feel that it is somewhat an abuse of learning to treat us, *apropos* of the modern application of one salt of antimony, to a discourse on the early contests respecting the employment of all preparations of this mineral; the extravagances of Basil Valentine and Paracelsus; the sarcasms of Guypatin; and the damnatory edict of the parliament of Paris.

In the first or historical part of M. Lepelletier's work, he, however, shews his good sense, by repelling the claim advanced by Hufeland of Rasori's *discovery*, (if we may so call it,) for Brendel and Schroeder, of the school of Goettingen, and in some degree for

Richter. If the mere employment of tartarized antimony in pectoral complaints, prior to the time of Rasori, is to deprive him of the credit due to him for introducing the practice, then may all British practitioners claim a share,—small certainly to each among so many;—for, from time immemorial, *small* quantities of this salt entered into the composition of the medicines given in this country in inflammatory diseases, those of the chest included. The essential features of Rasori's practice are, that the emetic tartar is always the principal, and not unfrequently the sole remedy; is given in very large doses, and with a view to an effect on the system and the disease, (Rasori calls this operation counter-stimulant,) entirely independent of any sensible evacuation produced.

If any writer can be justly regarded as the precursor of Rasori, it is unquestionably the late Dr. Thomas Marryatt, of Bristol, whose practice was in all essential points the same as that of the Italian professor: the antimonial salt was the sole remedy; it was given in large doses; and that the object was an effect totally exclusive of any sensible evacuation, is manifest from the following passage from the *eighth edition* of his Therapeutics, published at Bristol in 1788:* "I have seen many instances wherein a paper has been given every three hours, (of which there have been ten grains [of tartar emetic] in six powders,) without the least sensible operation, either by sickness, stool, sweat, or urine; and, though the patients had been unremittingly delirious for more than a week, with subsultus tendinum, and all the appearances of hastening death, they have perfectly recovered without any medical aid,—a clyster every other day excepted. I have lately seen a great many cases similar to the above, and the tartarized antimony has invariably produced the same effect." (*Ther.*, p. 7.)

We quote this passage with no disparaging view towards Rasori, but with a firm conviction that the practice he adopted and recommended, so many years after the publication of Marryatt's work, did not originate in any suggestion derived from it. Considering how long and generally tartarized antimony has been employed in medicine, with the intention certainly of producing a manifest evacuation, what more probable than that some individual should proceed to increase the dose, seeing that he could do so with safety and benefit to the patient, and should finally discover that, though the discharges ceased, the controlling power (a power with which we fully believe the salt to be endowed,) over fever and inflammation still continued! Is it not, moreover, not merely possible, but very probable, that the light which had thus gradually broken on one mind should, by a similar process, irradiate another? It is also but fair to Rasori to state, that Marryatt, although so strongly recommending the antimony in fever, and also in pleurisy, does not particularly direct it in pneumonia.

* The first edition of this singular work was published in Latin, in 1758.

The historical part of M. Lepelletier's work, from the time of Rasori's publication to the present day, contains much interesting matter, and nothing more interesting than the conflict of medical opinion it displays; but we would add, that the interest is not of a purely pleasurable kind. We are presented with the opinions of twenty-two medical men, all of considerable, and some of them of very high reputation; and a greater divergence of sentiment regarding what many might deem a very plain matter of fact cannot be conceived. Their testimony regarding the matter under investigation is recorded, in the majority of instances, by themselves, in two or three cases by others, and as witnesses they may be classed thus: eleven favorable, six unfavorable, and five neutral, or of mixed opinions. In the first class stand Rasori, Laennec, Peschier, Bang, Wolfe, Fontanelle, Téallier, Trousseau, Franc, Delpech, Lallemand; in the second, Strambio, Félix Vacquié, Dance, Rostan, Andral, Bouillaud; in the third, Lemasson, Maury, Recamier, Broussais, and Chomel.

Some of the numerical details are not less curious than the general clash of opinion. Laennec lost but two cases out of fifty-seven; Bang, of Copenhagen, but two out of fifty-four, treated with tartarized antimony; whilst the employment of the same method enabled Peschier, of Geneva, to treat pleurisy and peripneumony for five years, when they were the diseases he most frequently met with, and twice prevailed epidemically, without losing a single case. M. Bouillaud, on the other hand, states that M. Dauvin had furnished him with a document, by which it appeared that, of fifteen individuals treated by M. Louis for pneumonia with emetic tartar, six perished; and this appalling mortality of one in two and a half he contrasts with the results furnished by large bleedings, under which treatment he makes the fatality range from one in eight and a half to one in twenty.

We feel quite convinced that other circumstances, besides the difference of the practice adopted, had conduced to this prodigious discrepancy: indeed, this is manifest; for the disproportion in the mortality is nearly as two and a half to one in the comparison of groups of cases treated by the same method,—that of copious bloodletting; but we regret to say, that we find no specification of particulars by which a knowledge of these circumstances can be obtained, and the degree of their influence calculated. On the contrary, we observe, from the great mass of evidence collected from various quarters by M. Lepelletier, and which extends over nearly sixty pages, an almost total absence of the details which alone could give it any value. The weight of great names is pretty nearly equal on both sides, and the testimony (with the honorable exception of that of M. Laennec, who specifies age and other particulars with great minuteness,) appears utterly useless, excepting that it is calculated to impress our minds with the necessity of improving the method of observing and recording medical facts.

The scope and method of the practice of Rasori, as stated by himself, we now subjoin; premising, that many who approve of the treatment of pneumonia by large doses of emetic tartar, would describe by the term *large*, quantities much less considerable than those which he would thus designate. He embodies his plan on the following propositions: "1. To treat pneumonia, from its commencement to its close, by emetic tartar. 2. To make of this medicine the principal, and sometimes the sole curative means of this disease. 3. To diminish by its use the number of bloodlettings, and to be able occasionally to dispense with them altogether. 4. To administer this medicine in doses such as the boldest practitioners have never thought of employing; carrying the quantity to a scruple, a drachm, and even many drachms, in twenty-four hours. 5. To employ often many ounces of the salt during the treatment of the disease. 6. Finally, to be able to say with certainty that these powerful doses of the medicine produce neither vomiting nor abundant alvine evacuations; and that sweats take place only under the same circumstances as when the ordinary methods of treatment are adopted."

On the power of supporting these large doses of emetic tartar, to which the name of "*tolerance*" has been given, M. Rasori thus expresses himself: "I shall observe, in the first place, that the fitness of the living organism to support large doses of the salt, without producing vomiting or any other symptom of powerful action on the intestinal tube, belongs only to its morbid state, is limited to this, and lasts only so long as this. The general morbid state, which I designate by the word *diathesis*, is that which in all cases constitutes the fitness of the living body to support with impunity, or, to express it more correctly, with utility, as I shall afterwards shew, the different doses of the medicine. Peripneumony, like all severe diseases, has its increase and its *apogee*, after which it progressively diminishes, if it is to have a fortunate termination. The aptitude of the patient to support doses of emetic tartar more or less strong follows the same variations; that is to say, that it is less at the commencement of the disease, increases till this has arrived at its highest degree, and diminishes progressively with it. It is necessary, therefore, that the doses of the medicine should bear a relation to these variations; but, if they exceed the fitness of the body to support them, should this occur even at the height of the disease, we shall certainly then observe a repugnance to a remedy which before was taken with ease, or there will be nausea and vomitings: in other words, we shall discover manifest indications of what may be called the excessive action of the medicine. I rarely begin with less than twelve grains, to be taken in the course of the day, and I cause this dose to be repeated for the night: when I see that the pneumonia has already made some progress, I give at first a scruple, or even half a drachm, and afterwards I go on increasing daily to one drachm, or even many drachms, according to the morbid state."*

It appears to us that we shall be rendering a more essential service to the reader by a tolerably full commentary on this passage, in which the more valuable parts of the contents of M. Lepelletier's work may be incorporated with such information relative to the practice in question as may have fallen under our own observation, than by a formal analysis of a volume, which, professing to be a compilation, consists, almost necessarily, of every variety of fact and opinion, gathered from all sorts of quarters.

1. The doses recommended by Rasori are not those generally adopted in this country or in France. The reader will have remarked, in the analysis of the work of M. Louis, that, in his practice, the individual doses ranged from about a grain to nearly two grains; whilst the total quantity administered during the day was from six grains to twelve. M. Laennec, to whose authority and influence any popularity this remedy possesses in the more northern countries of Europe is mainly attributable, began with a single grain every two hours, till six doses were taken; the patient was then left to repose for seven or eight hours, if the symptoms were not urgent, and he experienced any inclination to sleep. In more severe cases, he gave the medicine uninterruptedly at the same interval and in the same doses, or sometimes increased the dose to a grain and a half, two grains, or two and a half.† M. Lepelletier's experience accords with that of M. Laennec: he states that the dose best supported by adults is comprised between the terms of six and twenty-four grains daily.‡ Our own method of administering it agrees nearly with the same authority; our doses ranging from one grain to two grains, and these being administered every two or three hours. The quantities given by Dr. Marryatt were, within a trifling fraction, of the same amount. The period during which it is necessary to continue the medicine must vary according to the circumstances of cases; but its extremes may be stated to be a single day and nine or ten days; the dose, if altered during the period, being so in an ascending, not a decreasing ratio.

If the question be asked, why this deviation from the practice of Rasori? we would reply, that practitioners of this country and France adopt the dose which answers their purpose, and feel unwilling to accumulate needlessly in the system a medicine which Foderé, Magendie, Cloquet, Serres, Christison, and other high authorities, have proved to be poisonous. It may be remarked, too, that we at least have not witnessed any case in which an unfortunate result under the employment of the medicine could be reasonably attributed to a deficiency of the dose. It is the fact,

* Rasori, quoted by Bayle, Biblioth. Thérap. t. i. p. 196; and Lepelletier, p. 12-14.

† Laennec, translated by Dr. Forbes, p. 256, 3d Edition; and Lepelletier, p. 17-18.

‡ Ibid. p. 168.

likewise, that phlogoses in the north of Europe are generally sporadic; whereas, epidemics of pneumonia and other inflammations are not unfrequent in Italy, which may account for Rasori and Tomassini having been obliged to adopt a more *heroic* practice than northern physicians have found it necessary to follow; epidemic being in general less tractable than sporadic diseases.

The vehicle employed is a matter of comparatively little moment, provided it does not act chemically on the salt. M. Louis used, as has already been mentioned, the distilled water of the flowers of the linden-tree; M. Laennec, infusion of orange-leaf. In this country, patients prefer the medicine in *pure* water, and distilled water is, for obvious reasons, to be preferred. Given in this way, it has very little taste; and may thus be administered in cerebral affections and maniacal cases, without the patient being conscious that he is taking medicine at all. An ounce of water to the dose is a suitable proportion.

2. Are we to give it alone, or combined with opium? The Italian practice is to give it alone. Laennec and Louis both combined it with opium. M. Franc, pupil of Professors Delpech and Lallemand, of Montpellier, relates a case in which the latter of these distinguished men administered emetic tartar, combined with syrup of poppies, in a case of rheumatism: delirium and a state of extreme agitation ensued. M. Lallemand being obliged to be absent, M. Delpech took charge of the case, and, by giving the antimonial salt without this combination, no distressing accident occurred, and a cure was speedily effected. (*Lepelletier*, p. 54.) In our adoption of the one method or the other, we should be guided by the same considerations as would influence us to withhold opium from the treatment of acute inflammatory disease, or to resort to its use. We do not, therefore, add opium to the emetic solution, when it is employed at the outset of a disease; but, when the tone and excitement of the system are in some degree broken, and when the local affection exists rather in a condition of distress than of imminent peril, to a patient rendered nervous and irritable by disease of some continuance, and by debility,—when, for instance, there is cough, pain, or restlessness, which is complained of,—we then conjoin laudanum with the solution, in the proportion of about five minims to each dose. Under these circumstances it allays painful feeling, without impairing the efficacy of the principal remedy.

3. To what extent does emetic tartar deserve confidence as the sole remedy, independent of bloodletting, in pneumonia and other inflammations? Rasori, the apostle of the practice, and who employed the salt most abundantly, as we have shown, mentions it, in the paragraph of his work which we have quoted, as a means only of diminishing the number of bleedings, and *in some cases* of dispensing with them. Laennec says, "as soon as I recognize the existence of the pneumonia, if the patient is in a state to bear venesection, I direct from eight to sixteen ounces of blood to be taken from the arm. I very rarely repeat the bleeding, except in the case of patients affected with disease of the heart, or threatened with apoplexy, or some other internal congestion. More than once I have even effected very rapid cures of intense peripneumony without bleeding at all; but, in common, I do not think it right to deprive myself of a means so powerful as venesection, except in cachectic or debilitated subjects. I regard bloodletting as a means of allaying for a time the violence of the inflammatory action, and giving time for the emetic tartar to act." M. Lepelletier, among the numerous pathological facts contained in his volume, gives from various sources the details of twenty-four cases of pneumonia successfully treated by venesection and tartarized antimony conjointly, and of twelve failures by the same method; of thirteen fortunate ones by tartar emetic, and of two fatal by the same means. Of cases of rheumatism, he furnishes us with five fortunate results, and six failures by bleeding and antimony; by antimony alone, cures eleven, failures eight. These numbers, it ought to be remarked, are not to be regarded as any evidence of the comparative efficacy of the two plans, M. Lepelletier having gathered cases of any kind from any quarter, where he could find them detailed with sufficient minuteness. The cases are, however, valuable from the accurate manner in which they are reported, and because they show how necessary it is in certain instances to apply both these powerful agents to the accomplishment of our purpose. This is forcibly evinced by the evidence of Rasori himself, who, in one case of pneumonia quoted, employed as many as thirteen bleedings; and in this necessity M. Lepelletier concurs. To advert again to our experience, we would remark that in pneumonia we have always, when about to adopt the antimonial practice, directed one full bleeding of twenty ounces or more at the commencement of our treatment. In bronchitis, and we regard it as a remedy equally efficacious in this disease as in pneumonia, we have often confided to it solely, even in cases of considerable acuteness; though, not unfrequently, where the constitution has sympathized strongly with the local affection, one moderate bleeding has been premised. In cerebral and meningeal inflammation, in which diseases we think highly of its powers, it has been used conjointly with bleeding. In the more sthenic form of delirium tremens, a form which occurs in young and vigorous subjects, in whom the constitution is not yet thoroughly sapped by habits of inebriety, we have employed it without bleeding, except by leeches applied to the scalp. In this disease we have found it singularly efficacious, producing calm where opium has not only failed to do so, but has menaced the patient with convulsions. In what may be termed the sub-sthenic form of the same disease, in which opium alone does not mitigate the agitation or produce sleep, and tartarized antimony alone is equally inefficacious, a grain and a half of the salt with twenty minims of laudanum every second hour, is invariably found to accomplish the object, after a few doses have been administered. This form of delirium tremens constitutes an exception to the rules we observe in the association of opium with the salt, both as to the period of the disease at which the combination is adopted and the quantity of laudanum given.

4. What Rasori has termed *tolerance* of the medicine is a question which has been a good deal discussed, and as there is a difference of opinion regarding it between such high authorities as Rasori himself and Laennec, we are not disposed to pass it unnoticed. The following are the facts of the case. When emetic tartar is given in the doses we have described, vomiting ordinarily follows the first, and generally, too, the second dose, the bowels being sometimes simultaneously affected, sometimes not; whilst the two or three doses next ensuing produce purging. After this, the alimentary canal becomes tolerant of the medicine, and no evacuation is produced: so far, indeed, from catharsis being the effect, it is often necessary to suspend the employment of the antimonial salt in order that a purgative may be administered. What is here stated is the general rule, and we need not dwell on exceptions. Rasori attributes this tolerance, in a paragraph already quoted, to what he terms the *diathesis*, in other words to the inflammatory condition of the system. M. Laennec ascribes it to the largeness of the dose, to habit, and to the agreeableness of the vehicle, conjointly. Those who would learn M. Lepelletier's opinion, and, at the same time, behold a plain question obscured by a cloud of words, and all the worst forms of scholastic ratiocination; and arguments bestowed on exceptions and idiosyncrasies, which ought to be reserved for the general rule; are recommended to peruse the chapter on the subject which will be found diffused from page 185 to page 199 of his treatise.

We agree with Laennec in the opinion that this tolerance depends on a concurrence of circumstances; and these we take to be, that, within certain limits, large doses of the salt are less emetic than smaller ones; habit, and the inflammatory diathesis. This latter circumstance, we think with Rasori, is the principal cause of the tolerance. In fact we find that an inflammatory condition of the system renders it capable of supporting agencies, besides the action of antimony, which in health would be most powerful and highly prejudicial. This is evinced by familiar facts regarding the operation of mercury, cathartics, bleeding, cold, &c. under the condition specified.

5. Are there circumstances which forbid our employing this remedy in diseases to which in other respects it is applicable? The only circumstance known to us which should forbid its employment, is the existence, in any disease, of gastric irritation. This point, too, has been the subject of controversy. M. Laennec does not consider the existence of gastro-enteritis as a contra-indication of the practice: nay, he says, that by means of it slight affections of that sort are dispelled. M. Broussais, as might be supposed, takes a contrary view, in which Lepelletier, Dr. Forbes,* and other authorities concur. Where such gastric irritation exists, we certainly would advise other remedies than the antimonial salt to be resorted to, or the gastric inflammation to be subdued by appropriate means before it is adopted. In a case of affection of the heart with violent bronchial inflammation, in which epigastric tenderness was simultaneously complained of, emetic tartar was administered. Not only was tolerance never established; but vomiting remained for some time after the salt was withdrawn, and was with difficulty subdued; whilst intense epigastric pain remained till the fatal close. In addition to the indications of cardiac and bronchial affections, the mucous lining of the stomach was found in some parts quite pulpy, and almost every where intensely vascular. It should be remarked, moreover, that the long-continued use of the salt induces redness and glazing of the tongue, and a sense of dryness and stricture of the fauces, which are tolerably clear indications that something of the same sort is, in all probability, existing in the stomach, since to its surface the tartarized antimony is so much longer applied than to that which is thus affected.

6. By what agency on the animal economy are the beneficial effects of this substance on certain inflammations produced? On this question, as on others, we have a host of opinions emanating from high authorities. Rasori, Tomassini, and Borda, consider it as a direct counter-stimulant, destroying the inflammatory diathesis. MM. Laennec and Duparque think it increases the activity of the absorbent system; whilst M. Téallier regards it as possessing a hidden curative property; an opinion in which M. Trousseau, on the whole, coincides. (*Lepelletier*, p. 180.)

So far as we have observed, we should consider its effects as an emetic to be of considerable benefit in certain diseases; in bronchitis, for instance, by clearing the air-passages of mucus; and in pneumonia and other inflammations the first evacuations, both by vomiting and stool, may not be without their advantages. Yet there is an agency independent of all this, and which appears after the tolerance is established.

There is a short chapter in M. Lepelletier's book concerning the ointment of Autenrieth (emetic tartar ointment), but it contains no information which is not perfectly familiar to all British practitioners. The application of the antimonial salt to the treatment of rheumatism is discussed at considerable length, but the corollary deduced from the facts collected is our best apology for not transferring them to our pages. "Emetic tartar," he says, "employed in the treatment of rheumatism, has, up to the present time, furnished only results of which the conclusiveness may be disputed,

* See note in Translation of Laennec, 3d Edition, p. 267.

but on which he must not yet definitely decide, science requiring other facts to pronounce irrevocably on this question at a future period." (P. 224.)

We certainly prefer the accurate observation, statistical method, and close induction of M. Louis, to the alien facts and more verbose reasonings of M. Lepelletier. It should be remarked, however, that the books of these two authors are in their scope and principle essentially different. M. Lepelletier's work is professedly a compilation of what he thought to be material facts, that he could find relative to his subject, with such deductions from them as he thought himself warranted in drawing. M. Louis not only frames his own inferences, but observes his own facts. Hence our opinion may be considered as rather an expression of general views as to the mode in which medical investigation should be conducted, than as a comparative estimate of the merits of the individual works. We recommend both to the reader; because we think an acquaintance with them calculated to improve his knowledge of that branch of therapeutics, the treatment of inflammation, which, though our stronghold, is far from the perfection it is destined, we hope, to attain. M. Louis' work is, moreover, worthy of his attention, not merely for the direct information it gives, but because it arms him with additional power to gather precise information for himself.

---

